# Cause of death in patients with newly diagnosed chronic lymphocytic leukemia (CLL) stratified by the CLL-International Prognostic Index

**DOI:** 10.1038/s41408-021-00532-1

**Published:** 2021-08-05

**Authors:** Yucai Wang, Sara J. Achenbach, Kari G. Rabe, Tait D. Shanafelt, Timothy G. Call, Wei Ding, Saad S. Kenderian, Eli Muchtar, Jose F. Leis, Amber B. Koehler, Susan M. Schwager, James R. Cerhan, Susan L. Slager, Neil E. Kay, Sameer A. Parikh

**Affiliations:** 1 Division of Hematology, Mayo Clinic, Rochester, MN USA; 2 Department of Quantitative Health Sciences, Mayo Clinic, Rochester, MN USA; 3 Department of Medicine, Stanford University School of Medicine, Stanford, CA USA; 4 Division of Hematology Oncology, Mayo Clinic, Phoenix, AZ USA

**Keywords:** Chronic lymphocytic leukaemia, Chronic lymphocytic leukaemia

Dear Editor,

Chronic lymphocytic leukemia (CLL) is an indolent B-cell lymphoproliferative disorder that is considered incurable [1]. The clinical presentation of CLL is heterogeneous and the rate of progression is variable. Many patients have an indolent disease course and can be observed for years, while a subset of patients experience rapid disease progression with symptoms and/or bone marrow failure requiring treatment. A recent advance in risk stratification has been the CLL-International Prognostic Index (CLL-IPI), which integrates major clinical and molecular prognostic factors into a single risk score. Specifically, CLL-IPI uses age, Binet or Rai stage, beta-2 microglobulin, immunoglobulin heavy-chain gene variable region (*IGHV)* mutation status, and *TP53* disruption status (17p deletion or somatic *TP53* mutation) to stratify patients with newly diagnosed CLL into four risk groups with distinct prognosis [2]. Subsequent studies have shown that CLL-IPI can predict time to first treatment (TTFT) and overall survival (OS) in newly diagnosed CLL [2–5]. Although CLL primarily affects older adults in whom comorbidities can be competing causes of mortality, we previously showed that CLL progression and CLL-related complications (including infections and second malignancies) were the leading causes of death in patients with newly diagnosed CLL, regardless of age and comorbidities [6]. In this study, we sought to investigate if the causes of death in patients with newly diagnosed CLL would differ according to the CLL-IPI risk group.

This study was approved by the Mayo Clinic Institutional Review Board. Patients diagnosed with CLL between January 1, 2000 and December 26, 2019 were identified from the Mayo Clinic CLL Database, which includes consecutive CLL patients who were evaluated within one year of diagnosis in the Division of Hematology at Mayo Clinic, Rochester, MN [7]. All patients provided an informed consent form. Clinical characteristics, follow-up data, and survival outcomes were abstracted from medical record. Cause of death was classified into one of four categories: CLL progression, CLL-related complications (infection or second malignancy), CLL-unrelated, and unknown, consistent with our previously published approach [6]. Cumulative incidences of cause-specific death were analyzed using Gray’s test, with deaths from different causes treated as competing events and deaths from unknown causes excluded. All *P* values were two-sided and considered significant if <0.05. Statistical analyses were performed using SAS version 9.4 (SAS Institute, Cary, NC, USA).

A total of 1274 patients were included. Baseline characteristics are shown in Table 1. The median age at diagnosis was 63 years (range 24–92), and 878 (69%) were male. Based on CLL-IPI score, 448 (35.2%) had low-risk disease, 442 (34.7%) had intermediate-risk disease, 317 (24.9%) had high-risk disease, and 67 (5.3%) had very-high-risk disease. The median follow-up time was 5 years (range 0–19 years). Five hundred and seventy-four patients received CLL treatment (Supplemental Table 1). The median OS was 17 years; and the OS rate at 5 years was 85% (95% CI: 83–87%) and 64% (95% CI: 60–68%) at 10 years. Of a total of 286 deaths, the cause of death was CLL progression in 99 (34.6%), infection in 16 (5.6%), second malignancy in 47 (16.4%), CLL-unrelated in 59 (20.6%), and unknown in 65 (22.7%) patients (Supplemental Table 2).

**Table 1:** Baseline characteristics of CLL patients included in this study.

	CLL-IPI low risk (0–1) (*N* = 448)	CLL-IPI intermediate risk (2–3) (*N* = 442)	CLL-IPI high risk (4–6) (*N* = 317)	CLL-IPI very high risk (7–10) (*N* = 67)	Total (*N* = 1274)
Age at diagnosis (years)					
Median	60	61	70	65	64
Range	(24–90)	(25–91)	(42–93)	(42–86)	(24–93)
Sex					
Female	161 (35.9%)	133 (30.1%)	79 (24.9%)	23 (34.3%)	396 (31.1%)
Male	287 (64.1%)	309 (69.9%)	238 (75.1%)	44 (65.7%)	878 (68.9%)
Rai stage at diagnosis					
0	335 (74.8%)	203 (46.0%)	46 (14.5%)	11 (16.4%)	595 (46.7%)
I	94 (21.0%)	163 (37.0%)	163 (51.4%)	30 (44.8%)	450 (35.3%)
II	17 (3.8%)	41 (9.3%)	47 (14.8%)	14 (20.9%)	119 (9.3%)
III	1 (0.2%)	7 (1.6%)	37 (11.7%)	3 (4.5%)	48 (3.8%)
IV	1 (0.2%)	27 (6.1%)	24 (7.6%)	9 (13.4%)	61 (4.8%)
Missing	0	1	0	0	1
CLL FISH, closest to diagnosis					
13q-	275 (61.4%)	165 (37.3%)	75 (23.7%)	2 (3.0%)	517 (40.6%)
Negative	115 (25.7%)	112 (25.3%)	74 (23.3%)	5 (7.6%)	306 (24.0%)
trisomy 12	45 (10.0%)	89 (20.1%)	91 (28.7%)	1 (1.5%)	226 (17.8%)
11q-	7 (1.6%)	67 (15.2%)	62 (19.6%)	1 (1.5%)	137 (10.8%)
17p-	0 (0.0%)	0 (0.0%)	13 (4.1%)	57 (86.4%)	70 (5.5%)
Other	6 (1.3%)	9 (2.0%)	2 (0.6%)	0 (0.0%)	17 (1.3%)
Missing	0	0	0	1	1
*TP53* disruption (*TP53* somatic mutation or 17p-)					
No	448 (100.0%)	442 (100.0%)	298 (94.0%)	0 (0.0%)	1188 (93.2%)
Yes	0 (0.0%)	0 (0.0%)	19 (6.0%)	67 (100.0%)	86 (6.8%)
*IGHV*					
Mutated	448 (100.0%)	116 (26.2%)	39 (13.6%)	5 (7.8%)	608 (49.0%)
Unmutated	0 (0.0%)	326 (73.8%)	248 (86.4%)	59 (92.2%)	633 (51.0%)
Missing	0	0	30	3	33

*CLL* chronic lymphocytic leukemia, *IPI* International Prognostic Index, *FISH* fluorescence in situ hybridization, *IGHV* immunoglobulin heavy-chain variable region gene.

For the entire study cohort, the cumulative incidence of death from CLL progression was 5.7% (95% CI 4.3–7.5%) at 5 years and 13.2% (95% CI 10.7–16.3%) at 10 years. The cumulative incidence of death from CLL-related complications (infection or second malignancy) was 3.2% (95% CI 2.2–4.7%) at 5 years and 8.5% (95% CI 6.5–11.2%) at 10 years, and the cumulative incidence of CLL-unrelated death was 3.8% (95% CI 2.7–5.3%) at 5 years and 8.7% (95% CI 6.7–11.3%) at 10 years (Fig. 1A).

**Fig. 1:**
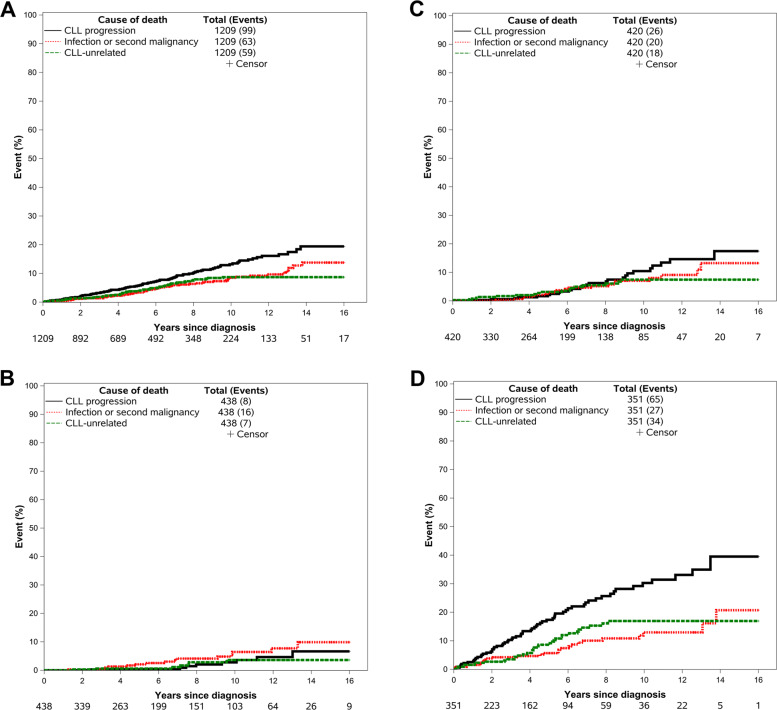
Cumulative incidences of deaths from different causes in patients with newly diagnosed CLL. **A** Cumulative incidences of deaths from different causes in all patients. **B** Cumulative incidences of deaths from different causes in CLL-IPI low-risk group. **C** Cumulative incidences of deaths from different causes in CLL-IPI intermediate-risk group. **D** Cumulative incidences of deaths from different causes in CLL-IPI high-/very-high-risk group.

For patients with CLL-IPI low-risk disease, the cumulative incidence of death from CLL progression was similar to that from CLL-related complications or CLL-unrelated causes, with a 5-year rate of 0.3% (95% CI 0.0–2.2%) vs 2.1% (95% CI 0.9–4.6%) vs 0.6% (95% CI 0.1–2.4%), and a 10-year rate of 2.8% (95% CI 1.1–6.7%) vs 6.4% (95% CI 3.7–11.3%) vs 3.6% (95% CI 1.7–7.7%), respectively (Fig. 1B). Similarly, the cumulative incidences of death from CLL progression, CLL-related complications and CLL-unrelated causes were comparable among CLL-IPI intermediate-risk patients, with a 5-year rate of 2.0% (95% CI 0.9–4.5%) vs 2.5% (95% CI 1.2–5.2%) vs 3.1% (95% CI 1.7–5.8%), respectively, and a 10-year rate of 10.4% (95% CI 6.8–16.0%) vs 7.0% (95% CI 4.3–11.5%) vs 7.4% (95% CI 4.6–11.9%), respectively, (Fig. 1C). In contrast, in patients with CLL-IPI high or very-high-risk disease, the cumulative incidence of death from CLL progression was higher than that from CLL-related complications or CLL-unrelated causes, with a 5-year rate of 17.3% (95% CI 13.1–22.9%) vs 5.7% (95% CI 3.4–9.4%) vs 8.6% (95% CI 5.7–13.1%), and a 10-year rate of 30.3% (95% CI 24.0–38.1%) vs 12.9% (95% CI 8.7–19.3%) vs 16.9% (95% CI 12.3–23.3%), respectively, (Fig. 1D). Patients with high-/very-high CLL-IPI risk group had higher cumulative incidence of death from CLL progression (*P* < 0.001), CLL-related complications (*P* = 0.013), and CLL-unrelated causes compared to the other groups (*P* < 0.001; Supplemental Fig. 1).

Our data suggest that the cause of death in patients with newly diagnosed CLL differs according to their CLL-IPI risk group. In patients with CLL-IPI low or intermediate-risk disease, the risk of dying from CLL progression is similar to that of dying from CLL-related complications (including infections and second malignancies) or CLL-unrelated causes. However, patients with CLL-IPI high- or very-high-risk disease have a ~3-fold higher risk of dying from CLL progression or associated complications such as infection or second malignancy compared to non-CLL-related reasons. Similar result trends were observed in follicular lymphoma (FL), another indolent B-cell malignancy, where risk of FL-related death was higher than risk of FL-unrelated death in patients with higher FLIPI (2 or 3–5) but not low FLIPI (0–1), highlighting the importance of prognostic index in informing the risk of death from different causes [8].

Results of our study have several implications in clinical practice. First, given the value of CLL-IPI in predicting TTFT, OS, and cause of death, diagnostic tests that determine the CLL-IPI risk group should be strongly considered in all patients with newly diagnosed CLL. From a patient counseling standpoint, it is important to discuss the heterogeneity of clinical outcomes and provide individualized counseling regarding survival. Historically, many patients were told that they were “more likely to die with CLL than to die of CLL”. Our data suggest that this is not true for patients with CLL-IPI high- or very-high-risk disease, which represent approximately one third of all newly diagnosed CLL patients in our study. Second, the increased risk of dying from CLL disease progression in CLL-IPI high-/very-high-risk groups suggest that improving CLL disease control is of utmost importance in these patients. Early intervention trials using novel targeted agents such as the CLL12 trial comparing ibrutinib to placebo [9] or the EVOLVE study (NCT04269902) comparing early vs. delayed venetoclax and obinutuzumab seem prescient in patients with high- and very-high-CLL-IPI risk groups [5]. Third, as 63/221 (29%) of deaths with a known cause were due to infections or second malignancies, there should be increased efforts to reduce the risks of the same, e.g., diligence in routine vaccinations and age-appropriate cancer screenings. Understanding the mechanisms of immune dysfunction and then developing strategies to restore more normal immune system in CLL patients are critical to improving their clinical outcomes. Finally, data from this study can be used in publicly funded health systems to guide decisions about obtaining molecular studies in patients with newly diagnosed CLL. Identifying patients with high- and very-high-risk CLL at the time of diagnosis may allow better counselling for such patients, an enhanced surveillance program for detection of progressive disease, and potential enrollment in early intervention clinical trials. On the other hand, testing to ascertain the CLL-IPI may provide reassurance to patients who are found to have low- and intermediate-risk disease.

The strengths of this study include a large prospectively followed cohort of consecutive CLL patients, comprehensive clinical information, follow-up duration, and competing risk analysis. The limitations include heterogenous therapies, ~2-decade time interval during which patients were studied when there were significant changes in treatments, and unknown cause of death in ~20% of the patients.

In summary, the CLL-IPI risk score has an important role in predicting causes of death in newly diagnosed CLL and has implications in improving patient counseling and advancing clinical practice.

## Supplementary information

Supplemental material

Checklist
