# Supplementary material for: Cause of death in patients with newly diagnosed chronic lymphocytic leukemia (CLL) stratified by the CLL-International Prognostic Index
Source: Blood Cancer J. 2021 Aug 5;11(8):140. doi: 10.1038/s41408-021-00532-1 (PMC8342603; doi:10.1038/s41408-021-00532-1)
Supplement: Supplementary file 1 — Supplemental material [file 41408_2021_532_MOESM1_ESM.docx]

**Supplemental Table 1**. **CLL-directed therapy for patients included in this study**

|  | **First line therapy**  **(n=574)** | **Second line therapy**  **(n=285)** | **Third line of therapy***  **(n=171)** |
| --- | --- | --- | --- |
| Chemoimmunotherapy^1^ | 236 | 78 | 27 |
| Other Chemotherapy^2^ | 85 | 42 | 34 |
| Monoclonal antibody therapy alone^3^ | 136 | 70 | 41 |
| Novel agents^4^ | 116 | 68 | 52 |
| Other^5^ | 1 | 27 | 17 |

*Median (range) number of treatments was 1 (1-12). Of those 171 who needed third+ lines of treatment, 152 had not received a prior novel agent. Of these, 83 received a novel agent at some point (44 as the third line, 39 as a later line), and 69 never received a novel agent.

^1^Chemoimmunotherapy (CIT) here refers to classical CIT regimens for CLL such as FCR, PCR, bendamustine-rituximab, and variants (e.g., FR, PR, CFAR, PAR, OFAR).

^2^Other chemotherapy included chlorambucil-based regimen, single agent purine analog or alkylating agent or combinations without a CD20 antibody, other rituximab containing regimens that are less frequently use for CLL (e.g., R-CHOP, R-C(V)P, R-CEPP, R-ICE).

^3^Antibodies used included rituximab, obinutuzumab, ofatumumab, ublituximab, alemtuzumab.

^4^Novel agents included ibrutinib, acalabrutinib, venetoclax and idelalisib.

^5^Other therapies included lenalidomide, everolimus, everolimus plus alemtuzumab, everolimus plus panobinostat, methotrexate, pembrolizumab, flavopiridol (alvocidib), CAR-T, DLI, etc.

**Supplemental Table 2. Summary of causes of death**

| **Cause of death** | **Number (n=286)** |
| --- | --- |
| **CLL progression** | 99 |
| **CLL-related complications** | 63 |
| **Infections** | 16 |
| Pneumonia | 7 |
| Sepsis | 3 |
| Other infections | 6 |
| **Second malignancies** | 47 |
| Lung cancer/Mesothelioma | 9 |
| Esophogeal/Colorectal cancer | 7 |
| Liver/Pancreatic cancer | 7 |
| AML/MDS/Myeloid malignancy | 4 |
| Squamous cell carcinoma (Neck/Larynx/Chest wall) | 3 |
| Bladder/Prostate cancer | 2 |
| Brain cancer | 2 |
| Cancer of unknown primary | 2 |
| Parotid gland cancer | 2 |
| Neuroendocrine tumor | 1 |
| Peritoneal cancer | 1 |
| Other cancers | 7 |
| **CLL-unrelated** | 59 |
| Cardiovascular disease^1^ | 19 |
| Cerebral/Neurological disease^2^ | 8 |
| Lung disease^3^ | 5 |
| Renal disease | 2 |
| Liver disease | 1 |
| Multiorgan failure | 1 |
| Natural causes | 2 |
| Others | 21 |
| **Unknown** | 65 |

^1^Coronary artery disease, congestive heart failure, cardiomyopathy, cardiac amyloidosis

^2^Stroke, encephalopathy, Parkinson disease, dementia

^3^Chronic obstructive lung disease, respiratory failure

Abbreviations: CLL, chronic lymphocytic leukemia; AML, acute myeloid leukemia; MDS, myelodysplastic syndrome

**Supplemental Figure 1. Cumulative incidences of deaths from difference causes in patients with different CLL-IPI risk groups.**

1. Cumulative incidences of deaths from CLL progression in patients with different CLL-IPI risk groups.
2. Cumulative incidences of deaths from CLL-related complications (infections or second malignancies) in patients with different CLL-IPI risk groups.
3. Cumulative incidences of deaths from CLL-unrelated causes in patients with different CLL-IPI risk groups.

**A**





**B**





**C**
